# Circulating collagen type I fragments as specific biomarkers of cardiovascular outcome risk: Where are the opportunities?

**DOI:** 10.1016/j.matbio.2025.03.001

**Published:** 2025-05

**Authors:** Emily M. Martin, Joan Chang, Arantxa González, Federica Genovese

**Affiliations:** a Nordic Bioscience A/S, Herlev, Denmark; b Institute of Biomedical Science, University of Copenhagen, Copenhagen, Denmark; c Manchester Cell-Matrix Centre, Division of Molecular and Cellular Function, University of Manchester, Manchester, UK; d Centre for Applied Medical Research (CIMA) Universidad de Navarra, Department of Cardiology and Cardiac Surgery, Clínica Universidad de Navarra, Department of Pathology Anatomy and Physiology Universidad de Navarra and IdiSNA, Pamplona, Navarra (Spain); CIBERCV, Instituto de Salud Carlos III, Madrid Spain

**Keywords:** Collagen type I, Biomarkers, Extracellular matrix, Cardiovascular risk

## Abstract

Collagen type I (COL1) is the most abundant protein in the human body and is a main component in the extracellular matrix. The COL1 structure vastly influences normal tissue homeostasis, and changes in the matrix drive progression in multiple diseases. Cardiovascular diseases (CVD) are the leading cause of mortality and morbidity in many Western countries; alterations in the extracellular matrix turnover processes, including COL1, are known to influence the pathophysiological processes leading to CVD outcome. Peptides reflecting COL1 formation and degradation have been established and explored for over two decades in CVD. This review aims to combine and assess the evidence for using COL1-derived circulating peptides as biomarkers in CVD. Secondly, the review identifies existing pitfalls, and evaluates future opportunities for improving the technical characteristics and performance of the biomarkers for implementation in the clinical setting.

## Highlights

• Collagen type I is overly deposited in fibrotic diseases, impacting organ structure and function.
• Biomarkers of collagen type I have been investigated in cardiovascular disease for decades, but the evidence for clinical use remains inconclusive.
• There are multiple mechanistic and clinical barriers that could influence consistency in cardiovascular indications.
• Improvement of collagen type I biomarkers could be achieved through a multivariable approach.

## Introduction

The extracellular matrix (ECM) plays an essential biological function by providing support for cellular and tissue organization and performance [1]. Collagens are the most abundant proteins in the ECM, comprising 30–70% of all ECM proteins dependent on tissue type, and are paramount to tissue homeostasis [[2], [3], [4]]. There are 28 collagen types, all containing a characteristic triple helical domain with Gly-X-Y repeats [5,6]. Within the heart, the ECM network and collagen homeostasis contribute to mechanical heart function and structural integrity [[7], [8], [9]]. Principally, the cardiac fibroblasts closely regulate the balance of type I collagen (COL1) and type III collagen (COL3) as the dominant fibrillar collagens, with the latter providing a more elastic phenotype [8,10]. COL1 is indeed the most abundant in the heart [11]. The triple helical domain of COL1 covers 96% of the whole protein, which is typically comprised of two α1 chains and one α2 chain, even though homotrimers ([α1]_3_) have been reported [5,6]. COL1 is a large interstitial and fibrillar collagen providing tensile strength and is critical to the structural network that the cells navigate through and function within. Recent studies highlighted that COL1 is synthesized, deposited and degraded under normal physiological conditions in a circadian-controlled manner to maintain tissue homeostasis [[12], [13], [14]]. Interestingly, in musculoskeletal tissues, it was shown that there is a dynamic “sacrificial pool” of collagen which acts as a buffer to protect the larger structures (the “permanent pool”) [[14], [15], [16]]. This tight homeostatic control likely extends to other collagen types and ECM molecules [14,17]. Multiple cell types and proteins coordinate the cardiac extracellular niche, which also acts as a reservoir for ECM-mediators, including matrix-degrading enzymes such as matrix metalloproteinases (MMPs) [7,18]. Dysregulation of ECM-mediating pathways leads to structural and functional changes, including the development of fibrosis and disease.

### Role of cardiac fibrosis in heart disease

Cardiovascular diseases (CVD) carry a steady burden to society, with one-third of all global deaths due to CVD, and it is mainly attributed to multiple modifiable risk factors and comorbidities such as hypertension, chronic kidney disease, diabetes, or smoking [[19], [20], [21], [22]]. The changes observed in the cardiac ECM and the resulting cardiac fibrosis are one of the major hallmarks of myocardial remodelling in patients with cardiac disease [23,24]. Sustained activation of profibrotic mechanism leads to interstitial fibrosis that significantly alters tissue architecture and homeostasis, contributing to cardiac dysfunction and failure [9]. For example, left atrial fibrosis is a predictor of adverse events such as recurrence of atrial fibrillation and cardiac arrhythmia caused by irregularities in the electrical activity of the atria [25,26]. Cardiac fibrosis is a highly complex and heterogeneous process that varies depending on the underlying triggering insult and the stage of evolution of disease. For instance, cell types, mechanisms and dynamic changes involved in the development of fibrosis after myocardial infarction differ from those found in pressure overload or in aging-related fibrosis [27].

Considering triggering mechanisms and localization, we can identify different forms of cardiac fibrosis such as: (i) replacement, (ii) reactive interstitial, and (iii) perivascular fibrosis, all characterized by an increased collagen deposition and myofibroblast activation [28]. The different types of myocardial fibrosis in situ are disease-dependent, and they can also be found in combination [28,29].

i. Replacement fibrosis is often seen in patients with acute organ injury, such as myocardial infarction and cardiomyopathies [28,30]. Tissue remodelling naturally follows post-injury [31,32]. After cardiomyocyte loss, immune cells infiltrate the area of injury to clear damaged cells and matrix proteins [33]. Matrix degradation enzymes are released to rapidly degrade collagen [31,34,35]. Fibroblasts are then activated in response to pathological stress and environmental stimuli, leading to overall collagen deposition being increased at the site of injury [33,35]. The formation of the scar causes myofibroblast persistence and ultimately the tissue becomes fibrotic due to the continued production of interstitial ECM proteins [35].
ii. Reactive interstitial fibrosis is more systemically driven by neurohumoral activation, or metabolic alterations, leading to collagen deposits build-up between myocytes [9,28].
iii. Perivascular fibrosis is localized around the cardiac vasculature and may contribute to vessel stiffness and to passive ischemia [8,36].

In patients with myocardial fibrosis of different aetiologies, the homeostatic balance between COL1 and COL3 is disrupted to favour a more stiff phenotype in which typically, COL1 synthesis is increased [9,37,38].

Notably, there is a plethora of collagens involved in the development and progression of cardiac fibrosis, including but not limited to COL3 and type VI collagen (COL6), from which cleaved fragments continue to be assessed in circulation through biomarkers [[39], [40], [41], [42]]. However, considering that COL1 constitutes 80–90% of the fibrillar collagen of the heart [11] and the extensive literature on COL1 and its associated biomarkers in CVD, we have focused on the evidence related to COL1 biomarkers.

### COL1 synthesis and degradation

**Fig. 1:**
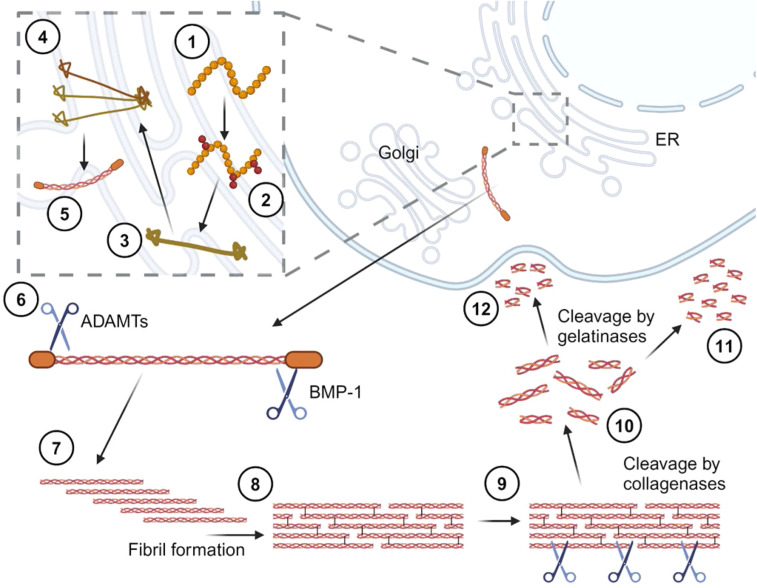
**Conventional process of COL1 synthesis and degradation.** Each chain of COL1 is translated at the ribosome (1) in the endoplasmic reticulum (ER) and post-translationally modified by hydroxylation (2). The N- and C-terminals are folded (3) and two alpha-1 and one alpha-2 chains are wound together from the C- to the N-terminus (4), using the chain-recognition sequence region. A complete and immature COL1 preproprotein is then exported from the ER, through the Golgi and released into the interstitial space (5). The propeptides are cleaved from the characteristic helical domain (6) specifically by ADAMTs at the N-terminus and BMP-1 at the C-terminus. Mature COL1 auto-aligns (7) and assembles into fibrils using cross-linking (8) stabilized by lysyl oxidase (LOX) and the LOX-like family. The processing of COL1 to a structural microfibril is complete (9). Afterwards, COL1 is cleaved by collagenases (10) and further cleaved by gelatinases (10). The peptides are either recycled back into the cell (12) or released from the tissue microenvironment (11), and into biological fluids, where they can be targeted as biomarkers. ADAMTs, a disintegrin and metalloproteinase with thrombospondin motifs; BMP-1, bone morphogenetic protein 1. Made in biorender.com.

Cardiac fibroblasts are the resident and primary collagen-producing cells of the heart [43]. Collagens are synthesized in the endoplasmic reticulum where preprocollagen chains are formed and undergo post-translational modifications (PTMs), including hydroxylation and glycosylation, into procollagen [44] (Fig. 1). PTMs such as hydroxylation and oxidation stabilise the collagen molecule, both at intra- and inter-molecular level, including thermal stability (prolyl and lysyl hydroxylases) and fibril crosslinking (lysyl oxidases) [45], while glycosylation may impact fibril diameter and crosslinking [46]. However, other types of PTMs, including cleavage products, and their potential impact on the structure and function of COL1 are still largely unelucidated [44,47]. In terms of formation, briefly, triple helical procollagen is wound before export from the cell, and C- and N- terminal propeptides are cleaved from the procollagen [44,48,49] (Fig. 1). Conventionally, propeptide cleavage occurs after cell exit; however, intracellular cleavage of the N-propeptide has been reported [50]. The cleavage of the C-propeptide, but not the N-propeptide, from the procollagen is essential for fibril formation [48,49]. The cleaved propeptides can be quantified by appropriate assays and be used as markers of collagen synthesis.

COL1 is tissue-dependently degraded by multiple MMPs and cathepsins, of both collagenase and gelatinase types, into small peptides and cleared by endocytosis [4,44]. Epitopes generated by protein cleavage and other PTMs, such as glycosylation and isomerization, can be termed neoepitopes [51,52]. Protein cleavage can release biologically active peptides, i.e. matricryptins or matrikines [44]. For example, the COL1-derived peptide “p1158/59″ is a matrikine that was associated to a reduction in left ventricular remodelling after myocardial infarction [53]. Quantification of COL1 fragments released during the processes of pathological tissue remodelling would help better understand COL1 turnover in various diseases and may facilitate better prediction, prognosis, and monitoring of disease.

## Current collagen type I biomarkers

### Current COL1 biomarkers for synthesis and degradation

Biomarkers can identify the molecular processes at a single individual's level, potentially determining or tailoring treatment to the person [54]. Circulating biomarkers are inexpensive and used to quantify biological changes at the protein level in serum and plasma, but there is a need for a larger variety of biomarkers for a deeper characterization of an individual's health [54]. To quantify ECM changes in pathology, an often forgotten but crucial alteration in the disease microenvironment, collagens or peptides of collagens could act as biomarkers. COL1 is central to the fibrotic microenvironment found in many chronic pathologies; hence, measuring biomarkers of COL1 turnover that can reach the blood stream and urine from the tissue of origin, could be useful to indirectly assess tissue remodelling processes and potentially inform on fibrosis activity.

**Fig. 2:**
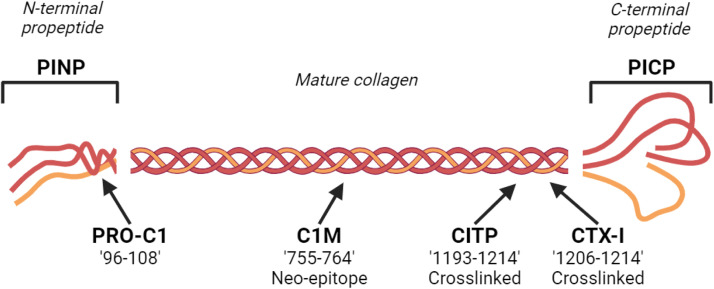
**Location of each commercial COL1 biomarker on COL1.** PINP and PICP are COL1 biomarkers representing the N- and C-terminal propeptides of COL1 cleaved during maturation. The PRO-C1 biomarker assay binds a specific 14 amino acid sequence of the N-terminal propeptide. C1M is a neoepitope specific degradation marker where the targeted sequence is cleaved by MMP-2, -9 and -13. The antibody used in the CITP commercial assay is specific to a location on the Col1a1 chain. CTX-I is a COL1 biomarker targeting crosslinked collagen at the C-telopeptide of COL1, where the antibody-targeting sequence is within CITP. All markers with a sequence defined are specified at the locations on the Col1a1 chain. Made in biorender.com.

To date, assays to quantify six different circulating COL1 fragments corresponding to different regions of the collagen have been developed and explored in relation to CVD (Fig. 2). The assays targeting the amino-terminal propeptide of COL1 (PINP and PRO-C1) and the carboxy-terminal propeptide of COL1 (PICP), produced by a disintegrin and metalloproteinase with thrombospondin motifs (ADAMTs) and bone morphogenetic protein 1 (BMP-1) respectively, measuring COL1 formation. A COL1 synthesis immunoassay can either quantify the trimeric or monomeric propeptide forms or only the trimeric form, depending on antibodies used for the assays [55]. The crosslinked carboxy-terminal telopeptide of COL1 released by the collagenase MMP-1 (quantified by CITP, also abbreviated to ICTP, target epitope ^1193^SAGFDFSFLPQPPQEKAHDGGR^1214^) is a marker for COL1 degradation [56,57]. CTX-I is a bone-specific biomarker derived from crosslinked COL1 fragments which is released by cathepsin K and can inform bone resorption by osteoclasts [58]. CITP and CTX-I share a similar target sequence at the telopeptide of COL1 which is a region required for fibril formation due to the site of crosslinking at this location, but the two biomarkers have been shown as having distinct enzymatic pathways of formation and clinical relevance [57]. The C1M assay targets another fragment of COL1, a neoepitope produced by MMP-2, -9 and -13 [59,60], and it has been mostly explored as a biomarker for inflammation-driven tissue degradation in rheumatologic diseases characterized by high inflammation [61,62]. Despite the availability of these six COL1 biomarkers, the clinical evidence for their use is still inconclusive. This article aims to collate and summarize the clinical data on these biomarkers in CVD.

### Successes and limitations of the collagen synthesis markers in CVD

The COL1 propeptides are targeted as markers for COL1 formation because each propeptide should be released in a 1:1 stoichiometric ratio to the newly mature COL1 molecule. However, it is not required that the N-terminal propeptide (quantified by PINP and PRO-C1) is released from the mature collagen for fibrillogenesis, potentially leading to a slower release rate for the N-terminal compared to the C-terminal propeptide (quantified by PICP) [48,49]. Further, the propeptides may also have a different half-life or dwell time in circulation, further complicating data interpretation. PINP and PICP levels have been mainly evaluated in heart failure (HF), and the evidence on the utility of COL1 formation markers in different CV manifestations are summarised in Table 1.

**Table 1:** COL1 formation biomarker evidence in cardiovascular diseases.

Condition	Biomarker	Change vs controls	Findings	Matrix	Assay	Reference
**Heart failure (undefined EF)**	PICP	Increased	PICP levels were not different between HFrEF and HFpEF patients.	Serum	MicroVue CICP EIA (Quidel, San Diego, California, USA)	[64]
	PICP	*NA*	PICP levels decreased in patients treated with spironolactone compared to placebo control after 1-month and 9-month treatment.	Serum	MicroVue CICP EIA (Quidel, San Diego, California, USA)	[89]
	PICP	*NA*	High PICP and low CITP:MMP-1 HF-patients have an increased risk of developing AF.	Serum	MicroVue CICP EIA (Quidel, San Diego, California, USA)	[129]
	PICP	*NA*	PICP associated cardiac abnormalities which improved after treatment with spironolactone mirrored in a reduction of PICP.	Serum	MicroVue CICP EIA (Quidel, San Diego, California, USA)	[149]
	PICP	Increased	PICP was reduced by treatment with torsemide for 8 months but not furosemide.	Serum	UniQ PICP RIA (Orion Diagnostica, Espoo, Finland)	[98]
	PICP	*NA*	PICP reduced by empagliflozin treatment at 12 and 52-weeks after start of treatment.	Serum	PICP EIA (Takara Bio, Shiga, Japan)	[82]
	PINP	*NA*	PINP associated with endpoint (cardiovascular death or HF hospitalization) when adjusted for major covariables.	Serum	ECLIA Cobas 8000 modular analyser (Roche Diagnostics, Basel, Switzerland)	[82]
	PICP	Increased	PICP is correlated to cardiotrophin-1.	Serum	UniQ PICP RIA (Orion Diagnostica, Espoo, Finland)	[63]
	PINP	*NA*	PINP was not predictive of all-cause or cardiovascular mortality in HF patients.	Serum	IDS-iSYS© (Immunodiagnostic Systems Ltd, Boldon, United Kingdom)	[150]
**Heart failure with reduced ejection fraction (HFrEF)**	PICP	*NA*	High PICP levels associated with increased risk of adverse CV outcome.	Serum	MicroVue CICP EIA (Quidel, San Diego, California, USA)	[76]
	PICP	*NA*	PICP independently related to B-type natriuretic peptide levels, left ventricular size and isovolumetric relaxation time.	Serum	MicroVue CICP EIA (Quidel, San Diego, California, USA)	[151]
	PINP	Increased	PINP reduced in sacubitril/valsartan treated patients compared to enalapril treated patients after 8-months.	Serum	UniQ PINP RIA (Orion Diagnostica, Espoo, Finland)	[93]
	PICP	*NA*	PICP identified a subset of patients that would respond to cardiac resynchronization therapy.	Serum	UniQ PICP RIA (Orion Diagnostica, Espoo, Finland)	[152]
	PINP	*NA*	Baseline PINP levels were not predictive of responders to cardiac resynchronization therapy.	Plasma (EDTA)	ECLIA Cobas 8000 modular analyser (Roche Diagnostics, Basel, Switzerland)	[135]
**Heart failure with preserved ejection fraction (HFpEF)**	PICP	Increased	After 1-year of eplerenone treatment PICP levels showed a reduced trend compared to the placebo group.	Serum	MicroVue CICP EIA (Quidel, San Diego, California, USA)	[65]
	PICP	*NA*	No change in PICP in HFpEF patients with type 2 diabetes treated with torsemide.	Serum	MicroVue CICP EIA (Quidel, San Diego, California, USA)	[153]
	PICP	*NA*	Reduction PICP in patients treated with spironolactone compared to placebo over 1-year.	Serum	MicroVue CICP EIA (Quidel, San Diego, California, USA)	[88]
	PINP	Increased	PINP unchanged in sacubitril/valsartan treated patients compared to valsartan alone.	Serum	UniQ PINP RIA (Orion Diagnostica, Espoo, Finland)	[94]
**Dilated cardiomyopathy (DCM)**	PICP	No change	PICP levels were not different between early and chronic DCM.	Plasma	Cloud Clone Corp. Houston, TX, USA	[69]
	PINP	No change	PINP levels were not different between early and chronic DCM.	Plasma	Cloud Clone Corp. Houston, TX, USA	[69]
	PICP	*NA*	No difference in PICP in DCM patients that did and did not suffer the combined endpoint of CV mortality and urgent hospitalization.	Plasma	Cloud Clone Corp. Houston, TX, USA	[85]
	PINP	*NA*	No difference in PINP in DCM patients that did and did not suffer the endpoint of CV mortality and urgent hospitalization.	Plasma	Cloud Clone Corp. Houston, TX, USA	[85]
	PICP	Increased	PICP increased in concentration over 12-months after diagnosis of DCM.	Plasma	Cloud Clone Corp. Houston, TX, USA	[79]
	PINP	Increased	PINP increased in concentration over 12-months after diagnosis of DCM.	Plasma	Cloud Clone Corp. Houston, TX, USA	[79]
	PICP	*NA*	Increased PICP levels from baseline to 12-months in DCM patients when stratified into grades of diastolic dysfunction.	Plasma	Cloud Clone Corp. Houston, TX, USA	[80]
	PINP	*NA*	Increased PINP levels from baseline to 12-months in DCM patients when stratified into grades of diastolic dysfunction.	Plasma	Cloud Clone Corp. Houston, TX, USA	[80]
	PICP	*NA*	PICP showed no association to late gadolinium enhancement or extracellular volume parameters.	Plasma	Bioassay Technology Laboratory, Shanghai, China	[81]
	PICP	*NA*	PICP levels were well correlated with late gadolinium enhancement and high PICP concentrations were associated with lower patient survival.	Serum	MicroVue CICP EIA (Quidel, San Diego, California, USA)	[78]
**Hypertrophic cardiomyopathy (HCM)**	PINP	No change	PINP showed no correlation to cardiac indices of diastolic dysfunction.	Plasma	Abbott, Abbott Park, IL	[73]
	PINP	*NA*	PINP was not associated with cardiac fibrosis or event rate.	Serum	UniQ PINP RIA (Orion Diagnostica, Espoo, Finland)	[72]
	PICP	No change	PICP showed no association to echocardiographic traits in overt HCM patients.	Serum	MicroVue CICP EIA (Quidel, San Diego, California, USA)	[71]
	PICP	Increased	PICP increased in HCM mutation carriers before development of HCM and, PICP increased in overt HCM compared to control.	Serum	MicroVue CICP EIA (Quidel, San Diego, California, USA)	[70]
	PICP	*NA*	PICP correlated with collagen volume fraction and PICP:CITP ratio is predictive of cardiovascular outcome.	Plasma	Elabscience, Wuhan, China	[84]
**Atrial fibrillation (AF)**	PICP	No change	PICP did not change between AF and non-AF patients who had CVD manifestations. PICP was associated to increased risk of AF in multivariate models.	Plasma	DuoSet ELISA (R&D Systems Inc., Minneapolis, Minnesota)	[83]
	PICP	Increased	PICP was higher in persistent AF compared to controls. PICP was highly associated with expression of NF-AT3 and NF-AT4 transcription factors.	Serum	Senxiong Biotechnology Industry Inc., Shanghai, China	[66]
	PINP	Increased	PINP levels were increased in persistent AF compared to controls. PINP was not associated with NF-AT3 and NF-AT4 transcription factors.	Serum	Senxiong Biotechnology Industry Inc., Shanghai, China	[66]
	PICP	No change	Within HF patients, a trend to increased PICP levels in AF patients compared to those in sinus rhythm and a mild correlation of PICP to left atrial diameter was observed.	Serum	MicroVue CICP EIA (Quidel, San Diego, California, USA)	[154]
**Myocardial infarction (MI)**	PINP	*NA*	PINP levels increased significantly from baseline to 1-month follow-up and steadily decreased through to month 9 in both eplerenone-treated and placebo groups.	Serum	UniQ PINP RIA (Orion Diagnostica, Espoo, Finland)	[91]
	PICP	*NA*	PICP increase from baseline to 9 month follow up was significantly lower in the eplerenone treated group compared to placebo.	Serum	MicroVue CICP EIA (Quidel, San Diego, California, USA)	[92]
**Hypertension**	PICP	Increased	Treatment with losartan for 1-year reduced PICP levels from baseline in hypertensive patients with severe fibrosis.	Serum	UniQ PICP RIA (Orion Diagnostica, Espoo, Finland)	[95]
	PICP	*NA*	PICP did not change from baseline to 8-months with torsemide or furosemide treatment.	Serum	MicroVue CICP EIA (Quidel, San Diego, California, USA)	[99]
**Population-based studies**	PRO-C1	Decreased	PRO-C1 significantly predictive of incidence of coronary artery disease event even when adjusted for multiple covariates.	Plasma (Heparin)	nordicPRO-C1 (Nordic Bioscience A/S, Denmark)	[86]

Heart failure (undefined EF) refers to research articles that do not define ejection fraction (EF). EIA, enzyme immunoassay; RIA, radioimmunoassay. NA: Not available.

#### Elevation of COL1 synthesis markers in disease

Multiple studies show that circulating levels of PICP are increased compared to healthy controls in multiple CVD, including HF (irrespectively of the left ventricular ejection fraction (EF)) [[63], [64], [65]] with no difference in PICP levels between HF with reduced EF (HFrEF) and HF with preserved EF (HFpEF) patients of hypertensive aetiology observed [64]. PICP is also increased in persistent atrial fibrillation (AF) [66]. In patients with HFrEF, those with AF did not have increased PICP levels compared to HFrEF patients in sinus rhythm, but PICP levels were correlated to left atrial diameter in all patient groups [67]. Most of the evidence focuses on the use of PICP as a diagnostic marker for cardiac disease, but PINP levels are also increased in patients with persistent AF compared to healthy controls [66].

Cardiomyopathies have diverse manifestations but commonly feature tissue stiffness and cardiac fibrosis [68]. COL1 formation markers have been assessed in cohorts of dilated cardiomyopathy (DCM) and hypertrophic cardiomyopathy (HCM). PINP and PICP showed no difference in DCM patients compared to healthy, between early-stage and chronic DCM patients [69]. Individuals can carry genetic predispositions to develop future HCM, and PICP levels have been shown to be increased in these mutation carriers against controls, possibly indicating a use of PICP as a marker of risk before clinical manifestation [70]. In the same study, PICP was increased in overt HCM patients compared to controls [70]. However, in the HCMNet study, the authors did not observe a change in PICP levels between overt HCM, preclinical HCM and controls [71]. For PINP in HCM patients, there was no association with fibrosis or with cardiac events found [72], nor a difference in PINP against controls [73]. The divergent results for the COL1 formation biomarkers in HCM could be due the patchy nature of characteristic HCM replacement fibrosis in the septum [74]. The inconsistent correlation may also be suggestive of an as-yet unexplored dynamic between PICP/PINP levels, propeptide clearance dynamics, and collagen fibril formation.

PICP was associated with left ventricle size and diastolic function in HFrEF patients, and it was also correlated with circulating B-type natriuretic peptide (BNP), a biomarker of cardiac injury [75]. Patients with an EF below 50% with low PICP levels presented enhanced cardiac reverse remodelling after 1 year of guideline-guided medical therapy (ventricular and atrial dilatation and EF) [76]. In addition, circulating PICP has been shown to be associated with myocardial fibrosis in HF patients [64,77] and in patients with DCM [78].

In DCM patients in different stages of disease severity, a steady increase in PINP and PICP over 12 months was observed [[79], [80], [81]], but without an association to cardiac fibrosis [79,81]. In idiopathic DCM, a moderate association of PICP with histologically assessed cardiac fibrosis was observed [78].

#### Prognostic usefulness of COL1 synthesis markers

PICP was independently associated to adverse outcome (cardiovascular death or hospitalization for HF) in HF patients from the EMPEROR study [76,82] and in a cohort of patients with HF and an EF below 50% [76]. The same association to outcome was observed with PINP in EMPEROR [82]. In the ARIC study, baseline PICP presented no difference in AF-developers or non-AF-developers at follow-up, but PICP was an independent predictor for developing AF after 11.8 years of follow-up [83]. The evidence of the relation of PICP with comorbidities was not evident in the EMPEROR study, while another study shows that PICP levels increased with the increased burden of comorbidities, including AF, anaemia and chronic kidney disease [76,82].

A single study found that the PICP/CITP ratio was a prognostic marker for cardiovascular outcome (new-onset AF, HF hospitalization, heart transplantation or death) in a small cohort of HCM patients [84]. In idiopathic DCM patients, PICP independently predicted a combined CVD endpoint (death, heart transplantation, HF hospitalization or life-threatening arrhythmias) [78] and when combining high PICP levels with a high late gadolinium enhancement (LGE) score, a cardiac magnetic resonance-based parameter for assessing replacement fibrosis, DCM patients had a lower event-free survival [78]. However, in another study, PICP and PINP were not prognostic for death or HF hospitalization, in DCM patients [85].

A single study reported PRO-C1, targeting an internal sequence in the N-terminal propeptide of COL1, as an independent predictor of major coronary artery disease (CAD)-related event (defined as fatal/non-fatal MI, or coronary revascularization) over 13 years in a Swedish population study [86]. PRO-C1 was significantly lower in the individuals that suffered a major CAD event compared to the control population [86]. The observed reduction in PRO-C1 is interesting because the cleavage of the N-terminal propeptide is not essential for fibril formation [87]; hence, although we would expect an increase in PRO-C1 in a more fibrous environment, this study hinted at a difference in collagen N-propeptide processing in CAD.

#### Therapy monitoring effects of COL1 synthesis biomarkers

COL1 formation biomarkers have also been evaluated as pharmacodynamic markers in response to treatment. Multiple treatments are used for the management of HF and other cardiac diseases including mineralocorticoid receptor antagonists (MRA), loop diuretics and angiotensin pathway targeted: angiotensin II receptor blockers (ARB), dual angiotensin receptor and neprilysin inhibitors (ARNi), and angiotensin-converting enzyme (ACE) inhibitors. The potential anti-fibrotic effect of these drugs has been evaluated using PICP in multiple studies. The Aldo-DHF and HOMAGE trials showed a reduction in PICP levels after treatment with the MRA spironolactone in patients with HFpEF diagnosis and in those at risk of developing HF, respectively [88,89]. Eplerenone treatment after MI, which induces massive ECM changes and can subsequently lead to HF [90], reduced PICP levels to a greater extent compared to the placebo treated group over 9 months in the EPHESUS study [91,92]. However, PINP levels increased within 1 month of MI and slowly declined after 3 and 9 months regardless of eplerenone or placebo treatment [91,92]. The EPHESUS study highlights the differential dynamics between PINP and PICP and resulting fibrotic responses. In the small EVEDES study, the MRA eplerenone did not greatly affect PICP or PICP:CITP levels after 1 year of treatment in patients with atrial switch repair for transposition of the great arteries, indicating a disease-dependent effect of MRAs on collagen turnover profiles [65].

The PARADIGM-HF study in HFrEF patients treated with sacubitril/valsartan (ARNi) showed reduced PINP levels compared to the ACE inhibitor enalapril treated group after 8 months [93]. However, in HFpEF patients (PARAGON-HF study), PINP levels were unchanged with sacubitril/valsartan dual treatment [94], potentially reflecting the increased fibrotic tissue profile at baseline in HFrEF patients compared to HFpEF patients.

The emergence of sodium-glucose cotransporter 2 inhibitors (SGLT2i), such as empagliflozin, has opened new therapeutic avenues in HF. In the EMPEROR trials, PICP levels were reduced by 5% at week 12 and by 8% at week 52 in empagliflozin-treated HF patients compared to placebo, indicating that empagliflozin may have a cardiac anti-fibrotic effect [82].

The ARB losartan showed a reduction in PICP levels and PICP:CITP ratio, but not CITP alone, in losartan-treated hypertensive patients after 1 year [95], which could be explained by losartan improving myocardial fibrosis by downregulating LOX expression and COL1 production, possibly through TGF-β1 (transforming growth factor β1) suppression [96,97]. Torsemide, a loop diuretic used for the treatment of hypertension and HF, reduced PICP levels over 8-months in hypertensive patients with chronic HF, and to a greater extent than the closely related furosemide [98]. However, these findings were not replicated in a larger study with a prolonged-release torsemide (TORAFIC), although these patients seemed to be in earlier stages of HF [99]. PICP was also used in a small biomarker-driven trial in HFpEF patients, the DROP-PIP study, to enrich the trial population and potentially to help reduce variability in treatment response with torsemide or furosemide, but without success, although a limited sample size (17 patients per arm) should be noted [100]. The evidence presented here for PICP and PINP as pharmacodynamic markers suggest that a treatment response may be dependent on the effect of said treatment on COL1 production by fibroblasts, and there may be a disease-specific effect.

### Why are there conflicting results with PICP and PINP?

Although cumulative evidence points to the usefulness of COL1 synthesis biomarkers in CVD, the results are in some cases inconclusive, both between studies and between different biomarkers within the same study. The technical differences between different immunoassays from various manufacturers could explain the variability of an individual biomarker across studies. For example, technical evaluation of the commonly used commercial PINP showed significant differences in a manual PINP assay compared to automated platforms, in particular the manual radioimmunoassay, which showed proportional bias compared to automated assays [55,101,102]. Despite the additional labour required for a manual assay and the increase in result variability, the Orion Diagnostica PINP is the only COL1 synthesis assay approved for clinical use by the FDA in the context of osteoporosis, and therefore, it is still a popular assay to use in the USA [55,101]. A recent report described highly variable biomarker concentrations in PICP and PINP assays across multiple cardiovascular indications [103]. Hence, based on the current assays available and studies measured, it is difficult to establish normal ranges and define cut-off values in CVD [103].

COL1 synthesis immunoassays either specifically quantify the intact propeptide in whole trimeric form or the total propeptide, including the trimeric and monomeric forms [55]. This is dependent on what the antibody (or antibodies) employed in the assay was raised against during development and the technical calibrator used for reproduction of the assay [55]. A potential difference introduced by the use of different matrices (serum or plasma) should also be considered, but strong correlations between the two matrices have been demonstrated at least for PINP [55,102]. It is vital to harmonize the different assays used, potentially with a correction factor based on the assay's technical calibrator [102], or at the least establish and report the technical differences of the commercial assays to help elucidate more conclusive evidence in CVD indications.

In most CVD studies, PICP is used as the COL1 formation biomarker, potentially due to the lack of association of PINP to most CVD indications. However, this also raises the question of why PICP and PINP levels can be so different when both measure COL1 formation. PICP and PINP vary in cleavage from the telopeptide, propeptide stability and biological activity, possibly explaining why PICP may outperform PINP as COL1 synthesis biomarkers in CVD. The molecular weight of PICP and PINP is 100kDa and 35kDa, respectively, with only PICP containing stabilizing interchain disulfide bridges [104,105]. Therefore, serological PICP may be more stable and slower to degrade than PINP allowing for more accurate measurement of the intact propeptide in PICP assays. The cleavage of the propeptides by different proteinases during COL1 maturation may also partly explain the lack of consensus between PICP and PINP evidence as predictors of disease [106]. Further, ADAMTs are inefficient enzymes which means that COL1 protomers retain some N-propeptides which can be tucked away and tolerated even in healthy tissues [107,108].

The consensus is that COL1 maturation is an extracellular process, involving the release of the full preprocollagen from the cell permitting cleavage to follow (Fig. 1). However, the N-terminal propeptide of COL1 can also be intracellularly processed via vesicular pathways [50,109], questioning to what extent PINP is released and the relative and measurable abundance of PINP compared to PICP. The intracellular processing of COL1 was thought to be specifically contained to the trans-Golgi network, however, pools of N- and C-terminal proteinases are located differently throughout the cellular secretory pathway [106]. The timing of intracellular propeptide cleavage has also been explored, such that PINP may be cleaved from the maturing collagen before PICP and that the cleavage of PICP is the final step to facilitate fibril formation at the cell-matrix surface [106], hence PICP would also represent COL1 fibril formation. It is unknown whether in this scenario the propeptides are released from the cell with the matured COL1 and further into the extracellular space of tissue and into the bloodstream.

Once trimeric propeptides reach circulation they are cleared differentially; PICP is cleared via the mannose receptor and PINP is via the scavenger receptor both on liver endothelial cells [110,111]. The half-life of PICP has been estimated as 6–8 minutes in circulation [111]. Hence, it could be questioned whether the total production of COL1 propeptides can be directly measured using PICP and PINP immunoassays. Another timing that should be considered is the circadian rhythm, where recent evidence indicated that COL1 synthesis, deposition and degradation is under circadian regulation, with PICP processing likely preceding COL1 maturation [13,14,106]. Interestingly, PINP in this study showed no circadian signature, although this may be due to a lower abundance of peptides available for identification, as PINP is much smaller than PICP [112]. The circadian variation shown in COL1 biomarker levels could explain the different results in different studies if samples were collected at different times. The discrepancy between PINP and PICP abundance in different diseases and treatment responses may suggest a yet-unknown dynamic relationship between the propeptides and collagen homeostasis.

Circulating PICP correlates with histologically assessed cardiac fibrosis [37,78]. At the tissue level, one study showed an association of PICP with the COL1 fibre abundance in the myocardium [63,113], while PICP in circulation was higher compared to that in myocardial tissue [113]. This suggests that COL1 biomarkers are also being released from additional sources, mostly likely from bone which is the major source of COL1 in the body [57,102]. The lack of tissue specificity of the COL1 propeptides may pose a problem, especially in patients with multiple manifestations. Nevertheless, a PICP gradient from the coronary sinus towards peripheral vein blood was detected in hypertensive HF patients [37], pointing to a contribution from the heart to circulating levels. Nevertheless, the large amounts of clinical data presented shows that a net increase in serological PINP and PICP is clinically relevant for the monitoring of CVD.

### Collagen type I degradation markers

To fully measure COL1 turnover, defined here as the formation and degradation of COL1, specific degradation markers are required. Quantification of COL1 degradation is important because it provides a measure of COL1 homeostasis systemically. ECM remodelling is a constant process but becomes altered as disease manifests, therefore measurement of collagen turnover could elucidate disease status in developing chronic disease and during acute injury response [52]. Table 2 summarizes the major findings of COL1 degradation markers in CVD.

**Table 2:** COL1 degradation biomarkers in cardiovascular disease.

Condition	Biomarker	Change vs controls	Findings	Matrix	Assay	Reference
**Heart failure (undefined EF)**	CITP	*NA*	CITP levels increase in patients treated with spironolactone compared to placebo control after 1-month of treatment.	Serum	UniQ ICTP RIA (Orion Diagnostica, Espoo, Finland)	[89]
	CITP:MMP-1 ratio	Reduced	Low CITP:MMP-1 levels were associated with high collagen crosslinking and increased risk of hospitalization for HF.	Serum	UniQ ICTP RIA (Orion Diagnostica, Espoo, Finland)	[127]
	CITP:MMP-1 ratio	*NA*	Low CITP:MMP-1 and high PICP HF-patients have increased risk of developing AF.	Serum	UniQ ICTP RIA (Orion Diagnostica, Espoo, Finland)	[129]
	CTX-I	*NA*	CTX-I was predictive of all-cause and cardiovascular mortality in univariate and multivariate analysis.	Serum	CrossLaps IDS-iSYS© (Immunodiagnostic Systems Ltd, Boldon, United Kingdom)	[150]
	C1M	*NA*	C1M was not associated with primary outcome (cardiovascular death or HF hospitalization) and levels did not change with empagliflozin treatment.	Serum	nordicC1M (Nordic Bioscience A/S, Denmark)	[82]
	CITP	Increased	CITP was increased in non-ischemic heart disease compared to ischemic disease.	Serum	UniQ ICTP RIA (Orion Diagnostica, Espoo, Finland)	[114]
**Heart failure with reduced ejection fraction (HFrEF)**	CITP:MMP-1 ratio	*NA*	The CITP:MMP-1 ratio was not associated to any clinical parameters or to outcome.	Serum	UniQ ICTP RIA (Orion Diagnostica, Espoo, Finland)	[76]
	CITP	*NA*	CITP was correlated to B-type natriuretic peptide (BNP) levels.	Serum	UniQ ICTP RIA (Orion Diagnostica, Espoo, Finland)	[151]
	CTX-I	*NA*	CTX-I did not predict response to cardiac resynchronization therapy, but a trend was observed.	Plasma (EDTA)	ECLIA Cobas 8000 modular analyser (Roche Diagnostics, Basel, Switzerland)	[135]
**Heart failure with preserved ejection fraction (HFpEF)**	CITP	Increased	In patients treated with eplerenone for 1-year, CITP increased to a lesser extent compared to placebo controls.	Serum	UniQ ICTP RIA (Orion Diagnostica, Espoo, Finland)	[65]
	CITP	Increased	CITP levels were increased in sacubitril/valsartan treated patients compared to valsartan alone 16-weeks after randomization.	Serum	UniQ ICTP RIA (Orion Diagnostica, Espoo, Finland)	[94]
	CITP:MMP-1 ratio	*NA*	Low CITP:MMP-1 and high PICP levels identify a subset of HFpEF patients with a higher risk of hospitalization and mortality.	Serum	UniQ ICTP RIA (Orion Diagnostica, Espoo, Finland)	[128]
	CITP:MMP-1 ratio	*NA*	CITP:MMP-1 ratio was unchanged in spironolactone treated patients compared to placebo over 1-year, but a high baseline ratio was associated with improved spironolactone-related improvement in diastolic function.	Serum	USCN Life Science (Houston, TX, USA).	[88]
	CITP	*NA*	CITP was inversely correlated to pulmonary arterial hypertension.	Plasma	HFN biomarker core laboratory (University of Vermont, Burlington,VT)	[115]
**Atrial fibrillation (AF)**	CITP	No change	A mild significant correlation of CITP with left atrial diameter was observed.	Serum	UniQ ICTP RIA (Orion Diagnostica, Espoo, Finland)	[154]
	CITP	Increased	PINP:CITP was an independent predictor for AF.	Serum	SRL (Tokyo, Japan)	[133]
	CITP	Increased	CITP was increased in HFpEF patients with AF compared to HFpEF patients in sinus rhythm	Plasma	HFN biomarker core laboratory (Burlington, VT, USA)	[155]
	CITP	No change	CITP levels did not change in AF patients prior to cardioversion compared to non-arrhythmia controls.	Serum	Cusabio Life Science (Wuhan, China)	[156]
	CITP	Increased	CITP levels were increased in AF patients during ablation procedure compared to non-arrhythmia controls.	Serum	Cusabio Life Science (Wuhan, China)	[157]
	CITP	*NA*	Strong correlation of CITP to cardiac extracellular volume fraction (CMR T1 mapping), as a marker of interstitial fibrosis	Serum	Cusabio Life Science (Wuhan, China)	[116]
	CITP	*NA*	CITP not predictive of AF recurrence.	Serum	Cusabio Life Science (Wuhan, China)	[158]
**Dilated cardiomyopathy (DCM)**	CITP	*NA*	CITP showed no association to late gadolinium enhancement or extracellular volume parameters (CMR-assessed)	Plasma	Serum CrossLaps ELISA (Immunodiagnostic Systems Limited, Boldon, UK)	[81]
	CITP	*NA*	CITP was not associated with cardiac collagen type I mRNA or with cardiac functional parameters	Serum	UniQ ICTP RIA (Orion Diagnostica, Espoo, Finland)	[117]
**Hypertrophic cardiomyopathy (HCM)**	CITP	Increased	CITP is increased in HCM patients compared to controls but only in plasma from the coronary sinus.	Plasma (EDTA)	UniQ ICTP RIA (Orion Diagnostica, Espoo, Finland)	[73]
	CITP	*NA*	CITP was not associated to late gadolinium enhancement or cardiac events.	Serum	UniQ ICTP RIA (Orion Diagnostica, Espoo, Finland)	[72]
	CITP	No change	No difference in CITP levels in overt HCM patients compared to levels in preclinical HCM patients was observed.	Serum	UniQ ICTP RIA (Orion Diagnostica, Espoo, Finland)	[71]
	CITP	*NA*	Ratio of PICP:CITP was predictive of cardiovascular outcome.	Plasma	Cusabio Life Science (Wuhan, China)	[84]
	CITP	No change	CITP was unaltered in HCM patients and HCM-at-risk patients compared to controls.	Plasma (EDTA)	UniQ ICTP RIA (Orion Diagnostica, Espoo, Finland)	[118]
**Myocardial infarction (MI)**	CITP	Increased*	CITP decreased in eplerenone-treated and placebo groups 1-month after MI which was further sustained until month 9. *against manufacturers reference range.	Serum	UniQ ICTP RIA (Orion Diagnostica, Espoo, Finland)	[91]
	CITP	*NA*	High CITP in ratio with COL3 and in combination with high B-type natriuretic peptide (BNP) was predictive of LV remodelling 1-year after MI.	Serum	UniQ ICTP RIA (Orion Diagnostica, Espoo, Finland)	[120]
**Coronary artery disease**	C1M	No change	C1M was not associated with extracellular volume (CMR assessed) in elderly patients with angina.	Serum	nordicC1M (Nordic Bioscience A/S, Denmark)	[121]
	C1M	*NA*	High C1M levels are predictive of CV events, CV mortality and all-cause mortality, when adjusted for age and diabetes.	Serum	nordicC1M (Nordic Bioscience A/S, Denmark)	[123]
**Hypertension**	CITP	No change	Treatment with losartan for 1-year did not change CITP levels from baseline in hypertensive patients with severe fibrosis.	Serum	UniQ ICTP RIA (Orion Diagnostica, Espoo, Finland)	[95]
**Population-based studies**	CITP	Increased	CITP was increased in patients who later developed HFpEF compared to those who did not develop HF. CITP was predictive of HFpEF development over 13 years.	Plasma (EDTA)	UniQ ICTP RIA (Orion Diagnostica, Espoo, Finland)	[124]
	CITP	*NA*	CITP was predictive of the development of AF over 10 years.	Plasma (EDTA)	UniQ ICTP RIA (Orion Diagnostica, Espoo, Finland)	[126]
	CITP	*NA*	CITP was associated with troponin-T and N-terminal pro-B-type natriuretic peptide.	Serum	UniQ ICTP RIA (Orion Diagnostica, Espoo, Finland)	[119]
	C1M	No change	C1M was not associated with MI or revascularization.	Plasma (Heparin)	nordicC1M (Nordic Bioscience A/S, Denmark)	[86]
	C1M	*NA*	High C1M levels were associated with increased risk of MI.	Serum	NordicC1M (Nordic Bioscience A/S, Denmark)	[122]

Heart failure (undefined EF) refers to research articles that do not define ejection fraction (EF). EIA, enzyme immunoassay; RIA, radioimmunoassay; CMR, cardiac magnetic resonance. NA: Not available.

#### Association of COL1 degradation markers with fibrosis and clinical parameters

The telopeptide of COL1 (CITP) is used as a COL1 degradation marker and is commonly used in parallel with PICP or PINP in CVD studies as a means of quantifying collagen turnover. Higher CITP concentrations were observed in ischemic versus non-ischemic heart disease patients and in patients with more advanced HF (according to NYHA classification), suggesting increased matrix remodelling in ischemic heart disease [114]. In HFpEF patients, CITP inversely correlated to right ventricular-pulmonary artery coupling, a measure of ventricular contractility and afterload [115]. Also, CITP was positively correlated with the extracellular volume, a cardiac magnetic resonance-derived marker for interstitial fibrosis, and significantly correlated to left atrial diameter in AF patients [67,116]. However, in DCM patients, CITP was not correlated with LV remodeling [117], nor with reactive or replacement fibrosis measured by extracellular volume or LGE, respectively [81]. Similarly, CITP levels in HCM patients showed no association to fibrosis or cardiac outcomes [72,73,118], but a significant gradient across the heart between simultaneous arterial and coronary sinus sampling was observed, suggesting increased COL1 degradation from the cardiac tissue [73]. CITP has been associated with markers for cardiac injury and stress, such as troponin-T and N-terminal proBNP at a population level [119,120].

Another degradation marker, C1M, showed no association to extracellular volume in symptomatic angina patients without significant CAD [121].

#### Prognostic value of COL1 degradation markers

COL1 degradation markers seem to be more relevant in ischemic heart disease including atherosclerosis and acute MI. The REVE-2 study in MI patients with low PIIINP (a marker of COL3 synthesis) to CITP ratio (or high CITP levels) showed an increased incidence of left ventricular remodelling at 1-year post-MI [120]. The neoepitope specific marker reflecting COL1 degradation by MMPs, C1M, was associated to the risk of developing MI in the PERF large general population study, but it was not predictive of reinfarction or death within 28 days of an MI [122]. In patients with atherosclerosis, C1M was associated with a higher risk of CV mortality and all-cause mortality over 6 years [123], but no association of C1M with CAD incidence up to 13 years was observed in the LSH population study [86]. The acute use of C1M in acute coronary syndromes has not been determined but could be interesting due to the rapid degradation of matrix proteins by MMPs in these conditions.

In the MESA population study, CITP levels were markedly higher at enrolment in patients who later developed HFpEF compared to those did not develop HF, and it was an independent predictor of incident HFpEF [124]. Hierarchical clustering showed that high CITP levels were strongly associated with a severe ‘pan-inflammatory’ phenotype of obese HFpEF patients [125]. Further analysis of the MESA cohort with a 10-year follow-up established CITP as a predictor for incident AF [126].

The ratio of CITP:MMP-1 has also been proposed as an indirect marker of collagen crosslinking in multiple studies [76,88,[127], [128], [129], [130]]. CITP is a cross-linked peptide, and cross-linking increases the resistance to degradation by MMPs, therefore for a given quantity of MMP, the higher the degree of cross-linking, less CITP will be released. Serum CITP:MMP-1 was inversely associated with cardiac collagen crosslinking in HF patients [127]. Chronic HF patients with a low CITP:MMP-1 ratio (suggesting increased collagen cross-linking) had an approximately 2-fold increased probability of rehospitalization for HF [127]. Furthermore, when combining CITP:MMP-1 levels with PICP values in hypertensive HF patients, those with a phenotype of high PICP and low CITP:MMP-1 presented a higher risk of rehospitalization for HF or cardiovascular death [131]. HF patients with low CITP:MMP-1 and high PICP levels had a higher prevalence of AF, a higher risk of new onset AF, as well as a higher risk of AF recurrence after ablation in AF patients [132]. However, in a large HFrEF study, no association of CITP:MMP-1 to prognosis was observed [76]. The CITP:MMP-1 ratio might be used as a surrogate to COL1 crosslinking in specific phenotypes of HF patients and facilitate some further patient stratification when used in combination with PICP.

The CTX-I assay, quantifying a crosslinked COL1 degradation fragment cleaved by cathepsin K, has been successfully verified as a bone resorption biomarker [57]. There is evidence to suggest that cathepsin K is an important COL1-degrading enzyme produced by the cardiovascular system, demonstrated by increased levels of cathepsin K and CITP in AF patients compared to healthy controls [133]. In HF patients, baseline CTX-I was predictive of all-cause and cardiovascular mortality over a median of 42.3 months [134]. However, CTX-I was unable to predict response to cardiac resynchronization therapy in HFrEF patients [135]. Whilst some evidence points towards the potential usefulness of CTX-I in CVD, wider approval for its’ use in CVD may be overshadowed by the success of the biomarker in aging-related bone diseases [136].

#### Investigating response to therapy with COL1 degradation markers

CITP has been investigated as a pharmacodynamic response biomarker primarily in studies testing the therapeutic efficacy of MRA, particularly spironolactone, in reducing fibrosis in CVD. The HOMAGE trial showed that CITP levels decreased after 1 month of spironolactone treatment in patients at risk of HF [89]. When modelled into a PICP:CITP ratio, where low PICP and high CITP (overall low PICP:CITP) potentially reflects increased collagen turnover, PICP:CITP was reduced at 1-month follow-up in spironolactone treated patients, and the reduction was sustained until final visit (9 months) [89]. A retrospective analysis from the HOMAGE trial defined good responders to spironolactone as those with low collagen crosslinking determined by a high CITP:MMP-1 ratio [130]. In the Aldo-DHF trial, which randomized HFpEF patients into treatment with spironolactone or placebo for 12 months, the high CITP:MMP-1 ratio was associated with a better response to treatment [88]. Aldo-HF and HOMAGE trials both showed that patients with high CITP:MMP-1 ratio (reduced crosslinking) at baseline improved left ventricular diastolic function with spironolactone treatment [88,130].

The effect of eplerenone, also an MRA, on acute collagen turnover was examined in the EPHESUS study, where patients were enrolled 3–14 days after an acute MI and randomized onto eplerenone and treated for 9 months [137]. At baseline, CITP was elevated before decreasing significantly over the first month of treatment but remained above reference controls [137]. High CITP levels could reflect the immediate release and breakdown of COL1 from the tissue during the inflammatory phase after acute tissue injury [137].

Overall, CITP has had less limelight compared to PICP and PINP in CVD. The individual CITP biomarker was shown to be elevated in multiple indications compared to controls and correlated to clinically relevant biomarkers and parameters. There is contrasting evidence, however, which may be explained by technical differences between immunoassays (even though the CITP assay is primarily sourced from Orion Diagnostica (Espoo, Finland; UniQ ICTP radioimmunoassay)) or by differences in cohorts and patient characteristics. CITP is cleared renally, and it is significantly influenced by kidney disease, therefore kidney comorbidities should be considered in CITP analysis [138]. Additionally, in literature, CITP is occasionally confused with CTX-I because they are targeting sequences in the COL1 telopeptide that overlap by 8 amino acids. Hence, effort should be made by vendors and researchers to report technical details more accurately.

## The need for new collagen type I biomarkers

### Current collagen type I biomarker barriers to overcome

The evidence for clinically useful existing biomarkers has taken decades to accumulate and should not be disregarded, but improvement and standardization of the available biomarkers should be prioritized. When critically appraising published data, several factors regarding the robustness of the results should be considered. In some cases, the divergence in biomarker results is due to unreliable assays. Therefore, it is critical for manufacturers to include all relevant technical information, including antibody design and production, specificity tests for the target sequence, and recording of intra- and inter-variations. The technical details for each assay are, in most cases, difficult to source through manufacturers, and variation in the assay should be better reported by investigators. This would allow for an in-depth technical comparison of the different assays, as performed with some COL1 markers [55,103]. Improvements or standardization of the methods can follow, as most of these assays have not been approved by regulatory agencies for clinical diagnosis. Additionally, standardized protocols for optimal sample processing would enhance the reproducibility of results. Lastly, the robustness of the study design should be carefully evaluated, considering factors such as sample size, appropriate statistical analyses, and adjustments for potential confounding factors, including kidney or liver disease.

A major limiting factor in using soluble COL1 biomarkers is the lack of organ specificity, partly due to the omnipresence of COL1 in organ systems, particularly in the skeletal system. Only a few papers have investigated organ-specific production of collagens, including aberrant COL1 homotrimers and a cardiac COL1-derived matricryptin [53,139,140], however, further research may show that the collagens or peptides of collagens originally thought to be organ-specific are more ubiquitous than first thought. Whilst it is possible to use the biomarker in stratifying patients with their clinical presentation, it would be ideal to find biomarkers with complete distinguishability between diseases, but this is difficult considering that PTMs are unlikely to be disease specific. The search for such collagen peptides is difficult due to abundant and analogous collagen proteinases circulating through multiple organs but finding differentially cleaved fragments of collagen may be a source of more specific COL1 biomarkers. Repeated measures of COL1 biomarkers over time may be more effective in tracking collagen levels, however, patient comorbidities would also need to be taken into consideration.

Another way to increase specificity and reduce variance is to determine biomarker panels that also include non-COL1 targets, which can be disease specific. For example, the CKD273 classifier used for chronic kidney disease utilizes 273 urinary distinct peptides, including but not limited to COL1 peptides, to form a single numeric value which is predictive of chronic kidney disease prognosis [141]. Importantly, a balance should be found between the complexity of the assay and providing an affordable, reproducible, fast and easy assay to be implemented in hospital laboratories. Therefore, there are multiple avenues for creating more advanced and targeted COL1 biomarkers, but considerable work is needed to fully understand them.

### Methods for identifying new targets for biomarkers

To develop novel COL1 biomarkers, specific targets need to be identified. Liquid chromatography coupled with tandem mass spectrometry (LC-MS/MS) is commonly used to sequence peptides creating large peptide databases, which are later analysed along with patient data to identify high abundance peptides associated with key clinical characteristics [142,143]. Similarly, capillary electrophoresis coupled to MS/MS is used and can better identify the smaller and highly charged peptides compared to LC-MS/MS [144]. Hence, the two coupled MS methods can be used to complement each other in identifying new biomarker candidates [144]. New methods to enrich collagen fragment detection in MS/MS techniques are emerging; for example, the use of dimeric collagen hybridising peptides that bind collagen fragments originating from the triple helical domain [145]. *In silico* methods using data pulled from public protein and secretome databases, have reduced the need for high throughput MS, however combining multiple methods would further improve biomarker candidate selection [146]. Nonetheless, navigating large peptide databases to identify a single biomarker is not trivial, therefore it is important to define strict criteria for candidate peptides. As novel targets are established and evaluated, there will be a need to target cleaved or post-translationally modified peptides highly specifically, which can be achieved through targeting neoepitopes.

### Advantages of targeting neoepitopes

Collagens are cleaved into many small peptides during normal degradative pathways, producing active signalling molecules (matricryptins and matrikines) and inactive fragments for protein recycling. The newly formed epitopes produced by cleavage and other PTMs can be termed neoepitopes, which can be targeted by specific assays [51,147]. Precisely raised antibodies binding the cleavage site of either a peptide originating from the mature molecule or of the propeptides, when implemented into an immunoassay format, can generate highly specific biomarkers. The main advantage of targeting neoepitopes is that, due to their size and antibody specificity, the sensitivity and specificity of the biomarkers is enhanced compared to large and difficult-to-target fragments of COL1 [147,148]. PTMs, including formation, degradation, glycosylation, isomerization and crosslinking, can be specifically quantified and compared to provide a complete COL1 profile [51,52]. Active matrix-derived signalling molecules, matricryptins and matrikines, can also be targetable and sensitively quantifiable with neoepitope-specific antibodies [147]. The quantification of these molecules would shed light on pathological mechanisms that go beyond collagen formation vs degradation.

## Conclusions

Cumulative evidence supports that the use of collagen-derived biomarkers for the assessment of COL1 metabolism and cardiac fibrosis holds promise for the future. These biomarkers could aid in screening, risk stratification, and therapy monitoring in CVD. While PICP is the most investigated COL1 biomarker, showing potential clinical usefulness mainly in HF, global employment of the current COL1 biomarkers in clinical studies has some limitations and challenges. Standardization of the commercially available markers would help to promote their implementation in the clinic, or alternatively, more specific cardiac COL1 biomarkers would be needed to improve disease-specific assessment.

## Data Availability

No data was used for the research described in the article.
